# Supplementary material for: Fano interference in quantum resonances from angle-resolved elastic scattering
Source: Nat Commun. 2021 Dec 13;12:7249. doi: 10.1038/s41467-021-27556-2 (PMC8668881; doi:10.1038/s41467-021-27556-2)
Supplement: Supplementary file 1 — Supplementary Information [file 41467_2021_27556_MOESM1_ESM.pdf]

Supplementary information

Fano interference in quantum resonances from angle-resolved elastic scattering

Prerna Paliwal[1†], Alexander Blech[2†], Christiane P. Koch[2*], Edvardas Narevicius[1*]

[1]Department of Chemical and Biological Physics, Weizmann Institute of Science, Rehovot 76100, Israel

[2]Dahlem Center for Complex Quantum Systems and Fachbereich Physik, Freie Universität Berlin, Arnimallee 14, 14195 Berlin, Germany

*Correspondence to E. Narevicius <edvardas.narevicius@weizmann.ac.il>, C. P. Koch <christiane.koch@fu-berlin.de>

[†]These authors contributed equally.

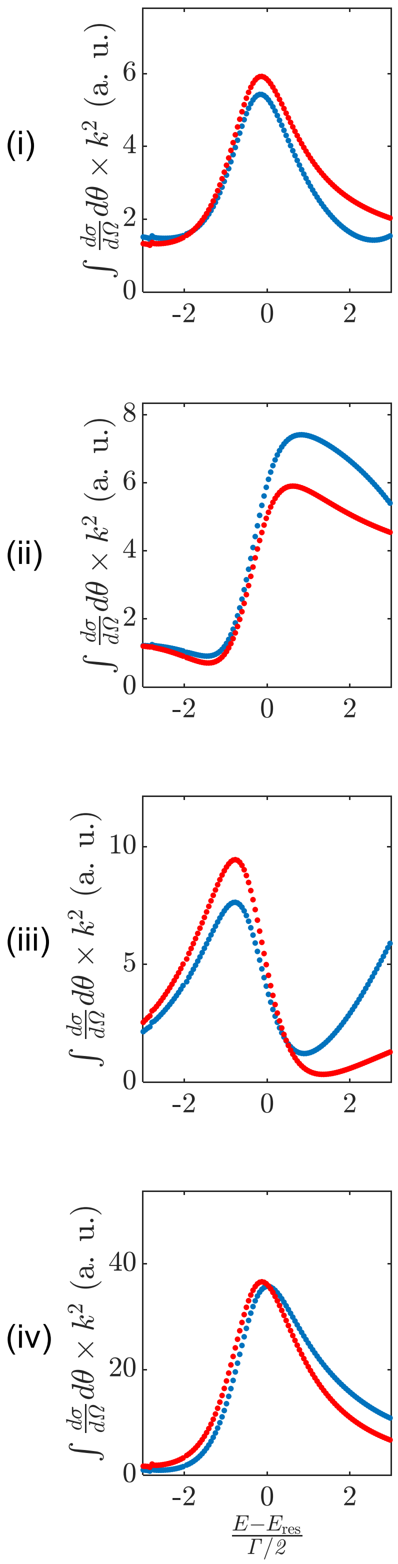

Supplementary Fig. 1| Resonant contribution from $l = 7$ in the vicinity of energy region dominated by $l = 6$. The theoretical angle-dependent cross section is shown including the contributions from all partial waves (blue) and omitting the contribution from partial wave $l = 7$ (red) to the cross section for the resonance at 4.8 K. The rising tail of the cross section observed in column (b), row (iii) in Fig. 2 of the main text disappears, indicating that it is caused by the higher energy resonance dominated by $l = 7$.

Anomalous line shapes observed in Fig. 2 (a) and Fig. 2 (b) of the main text for the angular section (iii): Here, we show why the rise in cross section at higher energies in Fig. 2 (iii) cannot be explained by our model. The main assumption in the derivation of our model are isolated resonance interacting with an energy independent background. When we have overlapping contributions from two or more resonances, the observed line shape cannot be explained by this simple model. In the case of angular section (iii), besides $l = 6$ resonance, another resonance dominated by $l = 7$ partial wave starts contributing significantly.

The Supplementary Fig. 1 shows the angle-resolved energy-dependent cross section obtained when the contribution from partial wave $l = 7$ is omitted and we observe that the rise of the cross section at higher energies vanishes for the (iii) angular section. Therefore, the increase in cross section observed in Fig. 2 (iii) of the main text at higher energies is caused by the rising edge of the resonance peak dominated by $l = 7$ [ref.[1]] At these energies, we thus have contributions from two different resonances, dominated by $l = 6$ and 7, respectively, which is not included in our model.

Supplementary References:

1. Paliwal, P. *et al.* Determining the nature of quantum resonances by probing elastic and reactive scattering in cold collisions. *Nat. Chem.* **13**, 94–98 (2021).
